# Protein Dynamics Governed by Interfaces of High Polarity and Low Packing Density

**DOI:** 10.1371/journal.pone.0048212

**Published:** 2012-10-26

**Authors:** Vladimir Espinosa Angarica, Javier Sancho

**Affiliations:** 1 Departamento de Bioquímica y Biología Molecular y Celular, Facultad de Ciencias, Universidad de Zaragoza, Zaragoza, Spain; 2 Biocomputation and Complex Systems Physics Institute (BIFI), Joint Unit BIFI-IQFR, CSIC, Universidad de Zaragoza, Zaragoza, Spain; University of Rome, Italy

## Abstract

The folding pathway, three-dimensional structure and intrinsic dynamics of proteins are governed by their amino acid sequences. Internal protein surfaces with physicochemical properties appropriate to modulate conformational fluctuations could play important roles in folding and dynamics. We show here that proteins contain buried interfaces of high polarity and low packing density, coined as LIPs: Light Interfaces of high Polarity, whose physicochemical properties make them unstable. The structures of well-characterized equilibrium and kinetic folding intermediates indicate that the LIPs of the corresponding native proteins fold late and are involved in local unfolding events. Importantly, LIPs can be identified using very fast and uncomplicated computational analysis of protein three-dimensional structures, which provides an easy way to delineate the protein segments involved in dynamics. Since LIPs can be retained while the sequences of the interacting segments diverge significantly, proteins could in principle evolve new functional features reusing pre-existing encoded dynamics. Large-scale identification of LIPS may contribute to understanding evolutionary constraints of proteins and the way protein intrinsic dynamics are encoded.

## Introduction

Protein dynamics range from local fluctuations of specific regions [1]–[3] to large-scale rearrangements involving partial or global unfolding of the native state [4]–[6]. Fluctuations between alternative structures within the native basin are thought essential for enzyme catalysis and protein recognition [1], [3], [7], while larger rearrangements may lead to protein misfolding and aggregation [4]. Dynamism and conformational variability are intrinsic to polypeptides and play a central role in protein folding and function [7], [8]. Besides, protein dynamics has been proposed to constitute an essential feature of protein evolvability [9]. Traditional views that the biological functions of proteins are carried out by single, well-defined conformations have been abandoned and there is mounting evidence that function is mediated by ensembles of alternative structures in equilibrium with the ‘native state’ [10]. Local structural fluctuations have been reported for some enzymes and promiscuous proteins in which multiple conformers contribute to binding a wide range of substrates or partners [1], [3], [7]. Remarkable flexibility involving wider rearrangements, and even fold transitions, has been described in some proteins where different folding species in equilibrium regulate their biological functions [5], [6], in prions that undergo a switch between the soluble and aggregated forms [3], or in proteins that tend to aggregate in specific conditions, causing severe diseases [4].

At present, the intrinsic flexibility and dynamic behavior of individual proteins can be investigated at atomic or residue level, in a one-to-one basis, by using well established techniques, such as hydrogen exchange NMR [11], [12], φ-analysis [13], [14], or Molecular Dynamics simulations [15], [16]. While these approaches have provided a wealth of information relating structure and dynamics, they are painstaking and cannot be easily applied on a proteome scale, nor can they reveal evolutionary relationships without extreme effort. Free energy estimation-based models, such as COREX [17], [18] are useful to predict local properties, such as hydrogen exchange rates [19]. The approach nevertheless requires extensive calculations and the estimation at residue level of a thermodynamic quantity, the free energy of folding, that is very difficult to calculate accurately even using careful parameterizations [20]. Coarse-grained computational models [21]–[24], such as Elastic Network Models [7], have proved very useful describing slow motions of proteins and have provided strong evidence that those motions are dictated to some extent by the fold geometry. These models, however, do not take into account specific interactions within the protein molecule and, therefore, can offer limited insight into the key physicochemical characteristics of highly dynamic protein *loci*. Thus, there is a need for simple and reliable methods of computational analysis that could help to identify and delineate the boundaries of such regions.

Proteins are generally organized into folding domains, some proteins consisting of just one. The interior of protein domains is well packed on average but significantly heterogeneous, such that tightly-packed regions, usually hydrogen bond-rich [25], coexists with others containing packing defects and cavities [26]. On the other hand, many important cellular processes are mediated by molecular recognition events occurring at protein surfaces that are constantly reshaped by internal motions. Since these motions should be governed by the relative stability of buried interfaces, we hypothesize here that domain cores will contain regions with physicochemical properties specifically suited to ease the reorganization of the contacting segments, hence allowing functionally relevant intradomain motions.

## Results

### Identification of LIPs by means of 3D-structure analysis

To test our hypothesis, we define protein interfaces as surface patches buried by the interaction of a contiguous protein segment of an arbitrarily defined length with the rest of the protein. Stable protein cores are characterized by a high content of hydrophobic residues, a fine matching of buried polar groups through hydrogen bonding, and tight packing. Most protein interfaces should, therefore, be highly apolar and the protein segments at the interface should be tightly packed. In contrast, protein interfaces involved in dynamics need to be intrinsically unstable and should display physicochemical features indicative of a low stability, such as a higher polarity and a lower packing density than stable interfaces.

To analyze the properties of protein interfaces, we have probed protein structures using a sliding-window approach. To that end, the three-dimensional structure of a given monomeric protein, as defined by a PDB file, is scanned from end-to-end using a contiguous peptide probe. For each peptide probe, two relevant properties of the probed interface are computed: the ratio of polar/apolar buried area, and the packing density (see methods). The corresponding computed values are assigned to the central residue of the probe. When the scanning is completed, property sequence profiles are built. The profiles so obtained are not very sensitive to probe length –*i.e.* 7-to-9-long probes give rise to almost identical profiles. However, short probes tend to make the profiles noisier while longer probes tend to average the properties of distant regions that may include both unstable and stable interfaces. We have thus set probe length to eight residues in all the cases reported. We have additionally tested whether the resolution of the structures could affect the outcomes of our method. Basically, the polarity profiles do not change significantly in the 1.2–2.8 Å resolution range (not shown), while the packing density profiles retain their shape –i.e. the position of maxima and minima– and exhibit slightly lower packing densities, which is in agreement with previous reports relating lower structural resolution with lower computed packing [27]. All together, our predictions based in the polarity and packing profiles are not sensitive to structure resolution.

**Figure 1:**
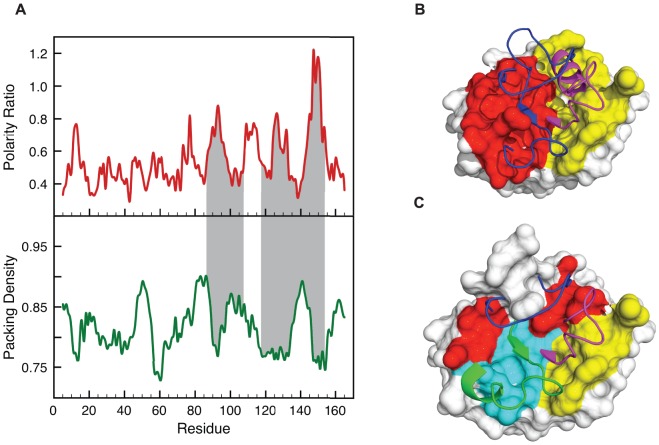
Identification and structural characterization of LIPs in apoflavodoxin. **A**) Stacked-aligned profiles for polarity ratio and packing density in *Anabaena* PCC 71191 apoflavodoxin (PDB: 1FTG, Resolution  = 2.0 Å). The property values (see definitions in Materials and Methods) are plotted against the position of the fourth residue of an eight-residue probe fragment. The segments encompassing residues 87–108 and 118–152, which have been found to be unstructured in the equilibrium intermediate of this protein [31], are highlighted in grey. We also show in grey dashed lines the polarity and packing cutoffs. **B**) Surface representation of buried atoms at interfaces 87–107 (yellow) and 118–152 (red) and the associated interacting fragments (in cartoon representation) colored purple and blue, respectively. **C**) Surface representation of the buried atoms according to our characterization of polar light interfaces (LIPs). The LIPs 87–99 (yellow), 120–133 (red) and 140–155 (cyan) are shown and the associated interacting fragments are colored purple, blue and green and are depicted in cartoon representation.

In Figure 1A we show the polarity ratio and the packing density profiles corresponding to a representative α/β protein: the apoflavodoxin from *Anabaena* PCC 7119. The polarity profile represents the ratio of polar over apolar surface area buried in the interface. A baseline with a polarity ratio of around 0.5 can be observed by visual inspection of the profile. Such a baseline is present in all polarity profiles we have built (see Figures S1 and S2 from the Supporting Material, for profiles of other representative proteins) so it appears to be characteristic of protein cores. To confirm that this is the case, we have computed using the ProtSA server [28] the polar and apolar solvent exposed areas in the folded state and in the unfolded ensemble [29] of a representative database [29], [30] composed by 19 proteins from different folding families and sharing less than 20% sequence identity. From these data (Table S1), we have calculated the polarity ratio characteristic of protein cores at 0.46±0.05. This value indicates that protein interfaces tend to bring into contact twice as much apolar atom surface than polar atom surface. Importantly, the apoflavodoxin polarity profile reveals protein segments that form interfaces of a much higher polarity than that of the baseline, one extreme being the interface centered at residue 150, where the contribution of polar atoms to the buried area is even larger than that of apolar atoms – i.e. polarity ratio >1. The packing density profile of the same protein (Figure 1A) also reveals significant local variations, with packing density minima centered at residues 13, 60, 92, 130 and 153.

Recent experimental work in our laboratory has identified the unstable regions of the apoflavodoxin from *Anabaena* PCC 7119 that experience local unfolding at mild temperatures, giving rise to an equilibrium intermediate [14], [31], [32]. The unstable regions correspond to residues 87–108 and 118–152, while the rest of the protein retains the native conformation in the intermediate. The two unstable regions of the protein are shadowed in grey in Figure 1A. These regions include the three peaks with higher polarity ratios. Noticeably, each of those peaks is mirrored by a minimum in packing density and thus represents a low-density (light) interface of high polarity. In the polarity profile, three additional, albeit lower and/or narrower, peaks of high polarity appear centered at residues 12, 77 and 102. It is clear that the peaks at 77 and 102 are not at density minima and cannot be defined as light polar interfaces. However, the one centered at residue 12 is at a packing minimum and represents an additional light polar interface. Indeed, this region, while ordered in the x-ray structure due to its association to a phosphate anion, appears disordered in solution even in the native conformation [31], [33].

**Figure 2:**
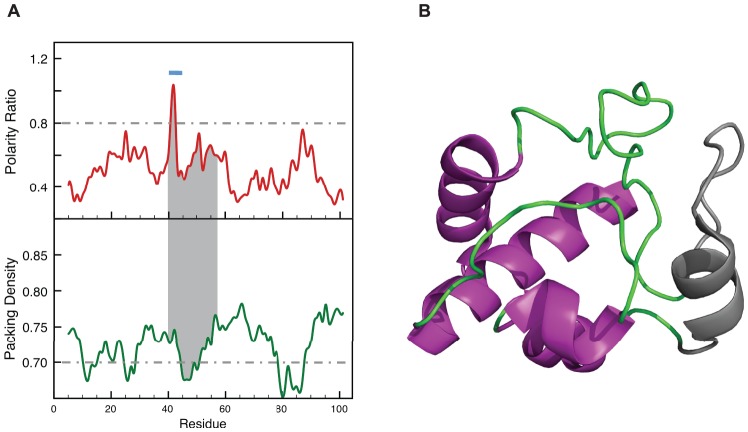
LIP and the lowest stability foldon in Cytochrome c. (A) Polarity ratio and packing density profiles of Cytochrome c (PDB: 1HRC, Resolution  = 1.9 Å). The segment shadowed in grey corresponds to the lowest stability region of the protein (infrared foldon: residues 40–57) according to equilibrium and kinetic H-exchange NMR experiments [39]. The light blue bar indicates the only LIP in Cytochrome c, which includes residues 40–45 and is located in the unstable foldon. (B) Ribbons representation showing the unstable foldon in grey. In the charts, the polarity and packing cutoffs are indicated as grey dashed lines

**Figure 3:**
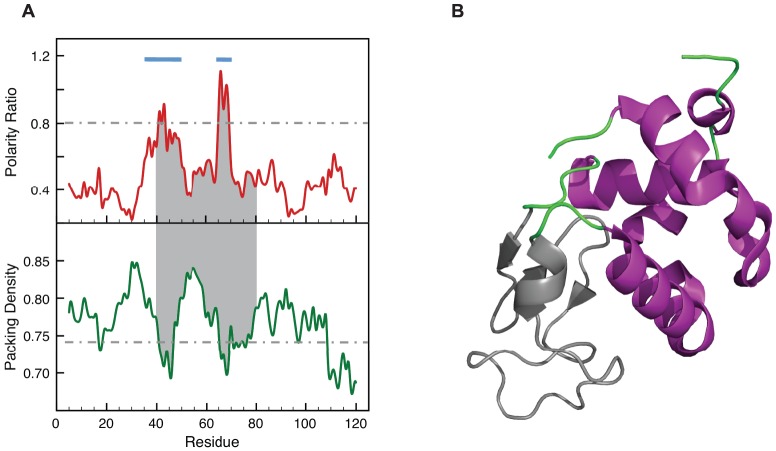
LIPs and the unfolded domain of the α-Lactalbumine Molten globule. (A) Polarity ratio and packing density profiles of α-Lactalbumine (PDB: 1HML, Resolution  = 1.7 Å). The segment shadowed in grey corresponds to the β-domain (residues 40–81), the one that lacks secondary structure in the molten globule intermediate [43]. The light blue bars indicate the two LIPs in α-Lactalbumine, encompassing residues 35–51 and 64–70, and essentially defining the β-domain. (B) Ribbons representation showing the unstable β-domain in grey. In the charts, the polarity and packing cutoffs are indicated as grey dashed lines.

**Figure 4:**
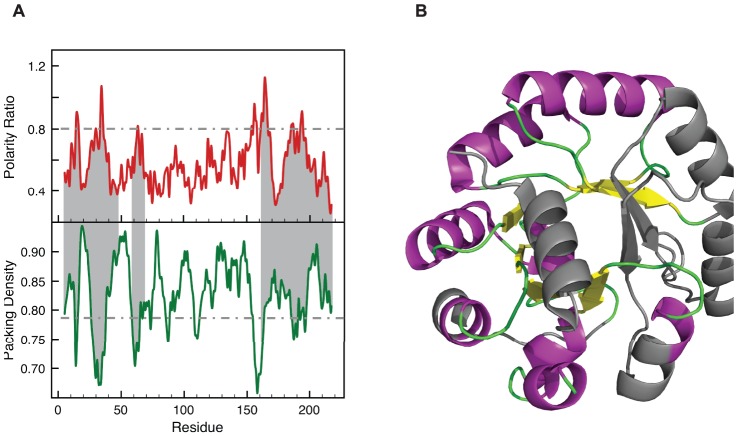
LIPs and the unfolded regions of the equilibrium (and kinetic) intermediate of Indole-3-glycerol phosphate synthase. (A) Polarity ratio and packing density profiles of Indole-3-glycerol phosphate synthase (PDB: 2C3Z, Resolution  = 2.8 Å). The segments shadowed in grey correspond to the unfolded regions of the equilibrium intermediate of chemical unfolding (intermediate Ia), which coincides with the on-pathway kinetic folding intermediate [44]. The light blue bars indicate the five LIPs in Indole-3-glycerol phosphate synthase. LIPs 7-18 and 23–40 map onto the N-terminal unfolded region of the protein (1–47). The next LIP, 58–68, defines the loop that is unfolded even in the native state (59–68). Finally, LIPs 148–170 and 178–205 are located at the C-terminal unfolded segment of the protein (162–220). (B) Ribbons representation showing the unfolded regions of the intermediate in grey. In the charts, the polarity and packing cutoffs are indicated as grey dashed lines

**Figure 5:**
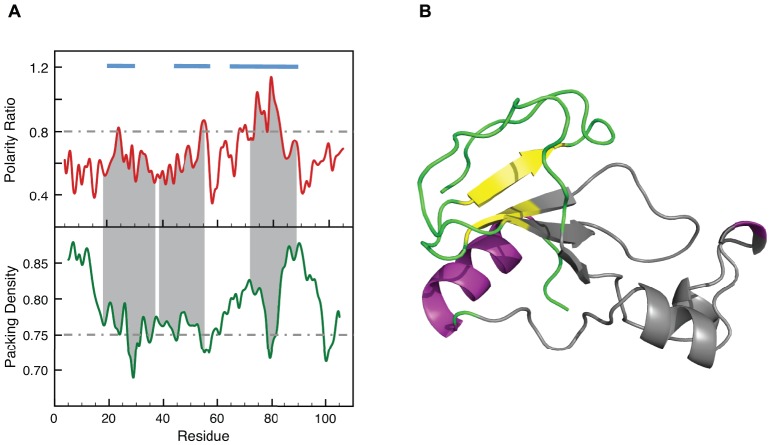
LIPs in the late transition state ensemble of barnase folding. (A) Polarity ratio and packing density profiles of barnase (PDB: 1A2P, Resolution  = 1.5 Å). The segments shadowed in grey correspond to the regions displaying φ-values equal to or lower than 0.5 in the late transition state of barnase folding (TS2) [47]. The light blue bars indicate the three LIPs in barnase: 20–30, 44–57 and 65–89. They closely correspond to the segments exhibiting low φ-values in the transition state (19–37, 39–55 y 72–88). (B) Ribbons representation showing in grey the transition state regions with low φ-values. In the charts, the polarity and packing cutoffs are indicated as grey dashed lines.

It is important to define light, polar interfaces in a quantitative manner so that the unstable regions of proteins can be predicted in an unbiased way. Since the properties calculated –*i.e.* polarity ratio and packing density– do not provide a value for the local unfolding free energy of the probe sequence, a threshold value must be defined to identify the unstable regions. Analysis of the solvent exposed area of the protein database [29], [30] used to compute the polarity baseline described above indicates (Table S1) that the polarity ratio of protein surfaces is of 0.75±0.08. This means that protein interfaces exhibiting polarity ratios of 0.8 or greater (0.8 rather than 0.75 is selected for simplicity) are more similar to surface regions than to protein cores. Based on this fact, 0.8 constitutes an appropriate threshold in the polarity profile and we propose that light polar interfaces can be identified as those organized around peaks exhibiting polarity ratios greater than this value. Each of these interfaces is considered to extend on either side of its polarity maximum to include the peak residues with polarity ratios above the 0.5 baseline (0.5 rather than the calculated average value of 0.46 is selected for simplicity). In all cases reported (see Figures 1, 2, 3, 4, 5) these interfaces appear located in packing density minima and, in fact, an anti-correlation between polarity ratios and packing densities is observed (Figure S3 and in the other examples studied in this work, not shown). While polarity ratios exhibited by protein interfaces or surfaces are expected to depend on general physical-chemical properties of the amino acid residues involved and on their relative abundances, there is evidence indicating that packing densities may be related to the specific fold and size of the protein [34], [35]. For the protein examples that will be discussed (Figures 1, 2, 3, 4, 5), the mean values of their interfaces packing densities are different. Thus, we define the regions of low packing density of a given protein as those with values below the mean of its specific density distribution minus two standard deviations.

For apoflavodoxin, only three peaks above the polarity threshold of 0.8 and located in low density regions are identified in this manner, the corresponding unstable segments being 87–99, 120–133, and 140–155. Since the experimentally determined unstable regions of apoflavodoxin are 87–108 plus 118–152 [31], the correlation between light polar interfaces and locally unstable regions is excellent for this particular protein. Figure 1B shows that although the unstable regions of apoflavodoxin are separated in the primary structure, they cluster together in the 3D structure and define a continuous unstable region. Comparison of Figures 1B and 1C allows noticing the structural correlation between the light, polar interfaces of the protein and the experimentally determined unstable regions.

### Occurrence of LIPs in all major protein classes

To refer to protein interfaces exhibiting a high polarity ratio and a low packing density we have coined the term LIP: **L**ight **I**nterfaces of high **P**olarity. The conservation of LIPs within structurally related proteins can be assessed using multiple sequence alignments to compare property profiles. Superimposition of the polarity ratio profiles corresponding to flavodoxins of known structure (Figure S1, top chart) indicates that they are very similar. The three key peaks characteristic of the flavodoxin from *Anabaena* PCC 7119 are present in the other flavodoxins. Similarly, comparison of the packing density profiles (Figure S1, bottom chart) indicates that the distribution of packing density heterogeneity in flavodoxin interfaces is also conserved, which means that light polar interfaces are conserved among flavodoxins.

Polarity and density analysis of interfaces present in a variety of proteins of known three-dimensional structure indicates that LIPs are present in all protein classes. Examples of conservation of polarity profiles in related proteins of classes α/β (folding TIM α/β barrel), α/β (folding Lysozyme-like), all α (folding Cytochrome c) and all β (folding Immunoglobulin-like β-sandwich) can be visually assessed in Figure S2. Conservation of the corresponding packing density profiles is similarly good in these folds (see Figure S4), which indicates that related proteins of a given fold share specific, conserved patterns of LIPs. We notice, however, that more distant proteins with the same fold can display different LIPs patterns, as it is the case for indole-3-glycerol phosphate synthase, Figure 4, and triosephosphate isomerase, Figure S2 panel A. Our results show that the polarity and packing density profiles are different for these two enzymes of similar sizes but belonging to two different superfamilies within the TIM α/β barrel fold and catalyzing rather disparate reactions such as decarboxylation and isomerization respectively.

### LIPs and intermediates at the native state basin and beyond

Tight packing, high hydrophobic burial and good pairing of buried polar groups are key ingredients of protein stability [36]. LIPs are bound to display high local instability due to their poor packing and low hydrophobicity, which, at least in apoflavodoxin, is associated to an abundance of buried polar groups not forming hydrogen bonds (not shown). Thus, LIPs are expected to experience transient local unfolding events from the native conformation more frequently than other regions and therefore to contain fast exchanging protons defining unstable foldons.

The correlation between LIPs and unstable foldons identified by their fast proton exchange rates can be illustrated by cytochrome c. The native basin of this protein has been characterized in detail by equilibrium proton exchange [37], [38]. Cytochrome c contains five foldons, or regions that can experience local unfolding uncoupled from that of the rest of the protein, that have been well defined at residue level. The more unstable one, so-called infrared foldon, comprises residues 40–57 [39]. The polarity and packing profiles calculated with our methodology for cytochrome c are shown in Figure 2. There is a single peak with polarity ratio higher that the 0.8 threshold, which defines a LIP spanning residues 40–45. Although not at the minimum center, this segment belongs to the wall of a deep packing density minimum including residues 40–55. Thus, the more unstable foldon in cytochrome c, with an unfolding free energy of 4 kcal/mol, contains the only LIP present in the protein.

Due to their low stability, LIPs are expected to become unfolded in solution conditions that are nevertheless compatible with the rest of the protein retaining the native conformation. LIPs should therefore correlate with the unfolded regions of equilibrium intermediates. These partly unfolded conformations tend to accumulate at moderately high temperatures or denaturant concentrations, or at extreme pH values, usually low pH. The apoflavodoxin intermediate discussed above is a fine example of the autonomous unfolding of LIPs in a thermal intermediate. The free energy difference between this intermediate and the native state is of just 1.5–2.0 kcal/mol [31], and the intermediate clearly belongs to the native basin. Not surprisingly, the LIPs in apoflavodoxin appear located in the functional regions involved in the binding of the FMN cofactor and of partner proteins [40].

A second common type of equilibrium intermediate is the molten globule [41]. Molten globules are typically observed after partial denaturation of certain proteins at low pH, although they have also been described in truncated proteins and in certain apoproteins at neutral pH. Molten globules have attracted attention because they bear similarity with kinetic folding intermediates and because they have been involved in physiological processes, such as membrane translocation. Structural characterization of molten globules is particularly difficult. One of the best-characterized molten globules is that of α-lactalbumine [42], [43], an α+β protein organized in two domains. Its molten globule retains a native-like secondary structure at the α domain, but not at the β domain encompassing residues 40–81. Inspection of the polarity and packing profiles of α-lactalbumine in Figure 3 clearly shows the presence of two LIPs centered at residues 43 and 66 and including residues 35–51 and 64–70. These LIPs make the β domain to be the more unstable one and contribute to define the residual structure of the molten globule.

A third common type of equilibrium intermediates is that found in chemical unfolding. The role of LIPs in chemical intermediates can be exemplified by the equilibrium intermediate accumulating in the urea unfolding of indole-3-glycerol phosphate synthase (IGPS) [44]. The equilibrium unfolding and the refolding kinetics of this protein have been extensively investigated by hydrogen exchange mass spectroscopy. The equilibrium intermediate accumulates at 5 M urea and consists of two conformations termed Ia and Ib. The more unstable species (Ia) is folded in the central segment (residues 48–161) and shows little or no protection in the 1–47 and 162–220 segments [44], [45]. In addition, the 59–68 loop appears disordered both in the native state and in the intermediate. The limits reported for the central folded and the N and C-terminal unfolded regions are approximate as they have been deduced from analysis of peptide fragments. The polarity and packing profiles of IGPS are shown in Figure 4. IGPS presents several peaks with polarity ratios higher than the 0.8 threshold that are located in packing density minima. Those centered at residues 15 and 34 define two contiguous LIPs spanning residues 7–18 and 23–40, which nicely correspond to the N-terminal unfolded region (1–47). The next LIP (towards the C-terminus) appears at residue 63 and extends on 58–68 in good correspondence with the loop that is unfolded in both the native and intermediate states (59–68). Finally peaks at 156 and 164, define a single LIP at residues 148–170, while peaks at 186 and 194 define an additional LIP spanning residues 178–205. These two C-terminal LIPs (residues 148–170 and 178–205) are in reasonable agreement with the C-terminal disordered segment of the protein defined from 162–220 [44], [45]. The structure of the IGPS equilibrium intermediate seems to arise as a consequence of the unfolding of the LIPs present in the native protein.

### LIPs and the protein folding reaction

The free energy difference between the IGPS equilibrium intermediate and the native conformation is of 8.5 kcal/mol [46]. This intermediate can hardly be considered to be within the native basin or be expected to display a functional role under native conditions. Interestingly, kinetic analysis of IGPS indicates that the structure of this equilibrium intermediate precisely corresponds with that of the on-pathway intermediate of the IGPS folding reaction (intermediate Ia) [44]. On the other hand, the infrared foldon of cytochrome c is also the latest folding region of the protein. Although LIPs have been defined as protein interfaces of the native conformation displaying low stability and therefore prone to experience local unfolding, it is possible that they also constitute late folding regions of proteins. Both the IGPS and the cytochrome c data point into this direction.

In addition to kinetic intermediates, a key species in protein folding reactions are transition states of folding. These ephemeral conformations are of high energy and can only be characterized by a combination of protein engineering and fast kinetics [13] or by computer simulations. Despite the large energy gap between transition state and native conformations, the available experimental information indicates that the differences are not so large at the structural level. We have thus investigated whether the not yet folded regions of transitions states could correspond to the LIPs of the native structure. One of the best-characterized transition states of protein folding is that of barnase. Recently, a combination of φ-analysis [13] and computer simulation was used to provide a structure of the transition state at the residue level [47]. The nativeness of the structure of a transition state around a given residue is described by its φ-value. Residues in a fully native or a fully unfolded environment in the transition state will show φ-values of 1.0 and 0.0, respectively. Barnase folds via a high energy intermediate and therefore two transition states appear in the reaction. The second transition state connecting the high energy intermediate with the native state is the one expected to be structurally closer to the native state and will be compared to the LIPs in the native structure. The polarity and packing profiles of barnase are shown in Figure 5. The segments of the protein exhibiting φ-values below 0.5 in the transition state (an arbitrary threshold selected to represent the more unfolded regions) are 19–37, 39–55 y 72–88, which are shadowed in grey in Figure 5. The barnase LIPs encompass segments 20–30, 44–57 and 65–89, which quite closely correspond to the regions with low φ-values [47].

### Assessing the statistical significance of property profiles

An important issue to take into account is trying to estimate the statistical significance of the observations reported in this work regarding the special characteristics of buried interfaces related to unstable protein regions. Although the polarity ratio profiles included in this study (Figures 1, 2, 3, 4, 5) visually show a clear correlation between the LIPs and the conformationally unstable regions at the sequence level, it would be interesting to supply statistical evidence of the differences between the values of polarity ratios obtained for those regions when compared to stable protein segments. We show in Table S2 the results obtained for a Mann-Withney-Wilcoxon rank-sum test for comparing the polarities of the buried interfaces of stable regions versus those of unstable ones and versus LIPs. These results demonstrate that the polarity of buried interfaces of unstable regions are statistically different from those calculated for stable ones in all the proteins analyzed. Table S2 also shows the expected fact that LIPs, as quantitatively defined above, display a significantly higher polarity than non LIP regions. As can be inferred from the low ℘*-*values for the comparison of the distributions returned by the test, the alternative hypothesis, determining statistical significant differences between the two distributions, should be accepted in all cases.

We also tried to compare our results with those obtained using a well-established software such as COREX [17], [48] in order to test the performance of the two methodologies when processing the same set of proteins. In Figure S5 the residue specific stability estimations calculated for the proteins included in this work using COREX is presented. A visual inspection indicates that for this kind of intermediates the predictions made by COREX for unstable regions do not correspond in some cases with the regions reported experimentally to be unstable. We repeated the statistical analysis described above to test whether the stabilities calculated by COREX for experimentally unstable regions were significantly lower than those corresponding to the stable regions of the proteins. The results from this test are included in Table S2 and prove that the alternative hypothesis determining significant difference holds only for Cytochrome c and indole-3-glycerol phosphate synthase. In these cases, the ℘*-*values obtained prove that the residue stability values of unstable regions are lower than those of stable regions. However for the three other proteins there are no statistical differences between the distributions. In all cases, the ℘*-*values obtained are higher than those obtained using our methodology. Not surprisingly, in only one of the five proteins tested (indole-3-glycerol phosphate synthase) there is a statistical correlation between COREX predicted unstable regions and LIPs.

## Discussion

On the hypothesis that the intrinsic dynamics of protein domains could be related to the presence of buried interfaces of low stability, we have devised a tool that allows to scan protein interfaces and to compute relevant physicochemical properties of the interfaces. Two key properties have been selected as indicative of low stability: the ratio of polar over apolar surface buried in the interface and the packing density at the interface. For a 200-residue protein it takes less than 2 minutes of CPU time in an average personal computer to calculate the polarity ratio and packing density profiles. Therefore, calculation of protein interface properties in a proteome scale is feasible. Our analysis indicates that protein interfaces display significant heterogeneity in polarity ratio and packing density. The protein examples discussed in this work contain interfaces, established by contiguous segments of 8 residues, whose polarity ratios vary from 0.3 to 1.2. In all proteins analyzed, a polarity baseline can be observed around 0.5, which appears to be the typical average polarity ratio of protein cores (Table S1). Above this baseline, peaks of higher polarity are observed. Since the average polarity ratio of protein solvent exposed surfaces is of approximately 0.8, the peaks with polarity ratios of 0.8 or greater identify the buried interfaces that are more polar than surface exposed regions (Table S1).

On the other hand, the packing densities vary from 0.65 to 0.9, with local minima along the profiles, but no obvious baseline value shared by the different proteins. Nevertheless, it is clear that most interfaces of high polarity ratio appear at packing density minima below the cutoff established. This can be quantitatively assessed by calculating for each particular protein its average packing density and then determining whether the peaks of high polarity display packing densities below that average minus two standard deviations. Such is the case of 13 out of 14 polar interfaces discussed in this work, the only exception being the red foldon of cytochrome c, which, as explained in the results section, appears at the wall of a deep minimum.

Proteins thus contain interfaces of high polarity and low packing density. We have termed them LIPs (Light, Interfaces of high Polarity), they are expected to exhibit low local stability and they can be easily identified. To test the hypothesis that LIPs are related to the structure of protein folding intermediates we have defined LIPs in a quantitative manner as interfaces including at least one residue with polarity ratio greater than 0.8 and extending to those flanking residues with polarity ratios greater than 0.5. In addition, the potential LIP should contain a clear minimum, defined as above, in the packing density profile.

The correspondence between LIPs, so defined, and the unfolded regions in protein equilibrium intermediates of different kinds is excellent. Figure 1 illustrates the correspondence of the LIPs in apoflavodoxin with the unfolded regions of the apoflavodoxin intermediate that accumulates in the thermal unfolding. Figure 4 shows the fine correspondence between the LIPs in IGPS and the unstructured segment of the equilibrium intermediate of its chemical unfolding. In Figure 3 we show the location of the α-lactalbumine LIPs in the β domain, the one deprived from secondary structure in the molten globule. LIPs also appear to correlate with unstable foldons exhibiting fast proton exchange from the native state and being late folding regions, as is the case of the infrared foldon of cytochrome c; see Figure 2. On the other hand the LIPs in IGPS also correspond to the not yet folded regions of its on-pathway folding intermediate, as can be seen in Figure 4. It is thus possible that, due to their instability, LIPs can only form on the scaffold provided by the rest of the protein. If this is the case, transition states of protein folding should also display conformations where the LIPs would still be essentially unfolded. The late transition state of barnase folding (Figure 5) illustrates this fact.

Altogether, our analysis reveals that protein domain cores contain interfaces of high polarity and low packing density that appear to be involved in protein dynamics, as they correlate with late folding events and with local instability in the native state that can lead to alternative partly unfolded conformations. Some of these conformations will be energetically distant form the native state, while others will be close in energy. The latter are expected to populate under native conditions and to get involved in function more easily.

As can be seen in Table S2, there is a strong statistical support indicating that the interfaces of unstable regions have a higher polarity than those of stable ones, which confirms that the physical-chemical characteristics of buried interfaces can be suitably used to identify conformationally unstable regions with a rather low error rate. The analysis of the polarity profiles in comparison with packing profiles indicates that the latest are less informative, as the fluctuations observed for the packing values are lower in comparison to those observed for interface polarity. This is why we used polarity as our primary source of discrimination. However, as can be seen from our results (Figures 1, 2, 3, 4, 5), the LIPs correlate in all cases with packing density minima, which is an interesting outcome in agreement with previous reports which had pointed to the relation of packing efficiency with local conformational changes and disorder [9], [49]. The reason why the statistical correlation between unstable regions and poorly packed ones is lower (not shown) is due to the fact that although unstable regions are indeed poorly packed, there are other poorly packed regions that are not particularly unstable, -*e.g* those exhibiting the characteristic low polarity of protein cores.

Importantly, the computational methodology developed here to identify these proteins' dynamic *loci* is simple and fast. A brief comparison with the COREX algorithm [17], [18] seems appropriate because both our structural method and COREX try to capture local differences in protein stability. COREX uses a more complex approach based in constructing an ensemble representation of the protein, which contains a large number of microstates. Since the method has to deal with a huge exponential search space, heuristic strategies are used to simplify the conformational search. Then, the stability of each residue is estimated by computing its free energy of folding from a parameterization of thermodynamic quantities as functions of the surface areas involved [50]. The method has proven to find correlation between calculated stabilities and hydrogen exchange rates [19]. In contrast, our method, does not attempt to provide stability evaluation at a residue level. We only try to identify the segments of the protein whose interaction with the rest of the protein is far from optimal. To achieve this goal we do not calculate free energy values, a very difficult task even if using careful parameterizations because, as it is known, free energy values are typically small and arise from compensation of much larger numbers involving enthalpic and entropic contributions. Instead, we compute simple physical-chemical and geometric properties to produce sequence profiles that help to highlight the regions of proteins displaying low local stability. We do not attempt either to give numbers for those stabilities. In this way, our approach is greatly simplified since our ensemble is linear with the number of residues in a given protein. Analysis of a 200-residue protein that takes less than 2 minutes with our method may take one day using the COREX server. One clear limitation of our method is that it does not provide stability values at residue level, however its performance identifying unstable regions related to experimentally characterized equilibrium and kinetic folding intermediates seems good (Figures 1, 2, 3, 4, 5). To evaluate the performance of COREX towards the same prediction targets we have run this algorithm, which is available at the COREX/BEST server: (http://best.bio.jhu.edu/BEST/index.php). The stability plots calculated for apoflavodoxin, cytochrome c, α-lactalbumine, indole-3-glycerol phosphate synthase and barnase are shown in Figure S5, where the experimentally determined unstable regions are shadowed in grey. Inspection of the figure indicates that for these particular types of intermediates COREX tends to provide a significant number of false positives (regions predicted to be unstable by COREX and not found to be unstable experimentally) together with some false negatives (experimentally unstable regions not predicted as unstable by COREX). The results included in Table S2, also prove that the stabilities calculated by COREX for unstable regions were not significantly lower than those corresponding to stable regions in three out of five of the proteins analyzed. In addition this statistical test shows that, in as much as it can be approximated by the ℘*-*values returned by the assay, the performance of COREX in distinguishing stable from unstable regions is lower than that of our method.

Although an analysis of the evolutionary significance of protein LIPs is clearly beyond the scope of this work, we would like to note some features of those buried interfaces that might turn out to be relevant. As we prove in this work, the methodology presented here is useful to identify unstable regions in proteins by means of our definition of LIPs, which in some cases match fairly well the location of unstable regions in proteins. Because those interfaces are simply characterized by displaying outlying values for averaged properties, their evolutionary conservation may not require a high conservation of the sequences involved. To illustrate this fact, structural multiple alignments of flavodoxin, cytochrome c and α-lactalbumine protein families are shown in Figure S6. The average protein identity percentage of these alignments ranges from 34% for flavodoxins to 50% for α-lactalbumin. Comparison of the alignments with the corresponding polarity ratio and packing density profiles obtained for members of those families (Figures S1, S2 and S4) shows that the profiles are conserved despite the sequence variation observed. On the other hand, the unstable regions of proteins studied in this work that superposed with LIPs often include protein segments with one side located at the interface and the other side exposed to solvent. Therefore, if the conservation at solvent exposed positions would tend to be lower than at buried ones, the solvent exposed backs of those interfaces could be suited to evolve new functions –*i.e.* recognition of new partners– because they could be mutationally tailored without seriously compromising the intrinsic dynamic nature of the interface. Actually, for the experimentally determined unstable regions and LIPs in Figure S6, the column averaged conservation scores estimated using the values obtained with CLUSTAL [51] for solvent exposed residues are roughly half the averages corresponding to buried residues (see legend of Figure S6). This means that in these regions buried residues presented to the interface and responsible for shaping its geometry and determining its physical-chemical characteristics are more conserved in average than residues in contact to solvent. These results demonstrate that the trends observed for the conservation of buried and exposed residues obtained for LIPs, which are a methodological definition to identify unstable regions, are shared by the stretches found to be unstructured experimentally. Finally, our preliminary analysis indicates that, as can be inferred from the comparison of the profiles in Figure 4 and Figure S2 panel A, within a given fold, less closely related proteins corresponding to different functional protein families could contain very different LIPs. It is thus possible that the different distribution of LIPs among distant homologues could help predict variations in dynamics, local stability and folding mechanism.

## Materials and Methods

### Estimation of buried interfaces' surface polarity

We have developed a set of *ad hoc* Perl and Tcl scripts to estimate, from the 3D structure of a given protein, the ratio of polar/apolar surface area of its buried interfaces. The input coordinates are used to extract a fragment of variable length –eight residues was the window size used–, then the cropped protein and the extracted fragment are processed using NACCESS [52] – with a Probe Size  = 1.40 – to estimate the surface area of the atoms buried by interaction of the two parts. The polar or apolar character of each atom type is set by the NACCESS library, and it is attributed to its surface. Using this information we defined the polarity ratio (PR) as follows:
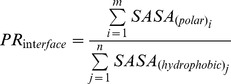
in which the total area of polar atoms as defined by NACCESS is divided by the total area of apolar atoms at the interface. This procedure is repeated by means of a sliding-window approach that permits the generation of a *PR*_interface_ profile of all interfaces along the structure of a protein. In these profiles, the PR of each interface appears assigned to the fourth residue of the 8-residue probe. 7-residue or 9-residue probes give rise to close to identical profiles (not shown).

### Interface packing density

We have computed the packing density of buried interfaces (*ρ_interface_*) using the following expression:
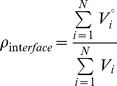
in which the numerator corresponds to Voronoi standard atomic volumes and the denominator to the real Voronoi atomic volumes of the atoms found at the interface. The standard volumes were derived in a recent report from an extensive study of the intramolecular contacts made by atomic groups in small molecule crystals [26]. The actual Voronoi volumes of the atoms at the interface were calculated using the program CALC-VOL [53]. Packing density values close to 1 correspond to tightly packed interfaces. The iteration of this calculation along the structure of a protein generates the packing density profiles presented in this study. In these profiles, the packing density of each interface appears assigned to the fourth residue of the 8-residue probe. As in the case of the Polarity profiles there are no significant differences in the packing density profiles obtained for 7- and 9-residue windows.

### Structural multiple alignments

The structural alignment of members of different protein families were constructed with the Multiseq package of VMD [54], based on the STAMP algorithm [55]. We aligned all the structures available for the flavodoxin, cytochrome c and α-lactalbumine and the resulting structural alignments were processed with JOY [56] and CLUSTAL [51] to include information concerning residue surface exposure (see Figure S4). For the other protein families studied in this work the small number of members with solved structure precluded building an informative enough structural alignment.

### COREX local stability calculations

The structures of the five proteins analyzed in this work (apoflavodoxin, cytochrome c, α-lactalbumine, indole-3-glycerol phosphate synthase and barnase) corresponding to different representative folds were processed with the software COREX [17], [48] available at (http://best.bio.jhu.edu/BEST/index.php) to estimate the local stability of protein regions. For each protein, a structure ensemble was first generated, which is used by the program in subsequent calculations. Based in the ensemble of structures generated, entropy-weighting factors were determined and stability constants calculated. The results obtained are represented in Figure S3.

### Assessing the statistical significance of the profiles

In order to evaluate the significance of our results we performed a non-parametric test to compare the polarity ratio of buried interfaces adjacent to structurally unstable regions and of our predicted LIPs with conformationally stable protein segments or with non LIP regions, respectively. For each polarity profile we performed a one-sided Mann-Withney-Wilcoxon rank-sum test with a confidence interval of ℘<0.05 to test the significance of obtaining higher buried interface polarities in unstable segments confirmed experimentally or in LIPs, when compared to the polarities of stable regions, see upper half of Table S2. In order to make a quantitative comparison of our methodology with COREX [17], [48] we performed the same statistical test for the residue specific stability profiles obtained with this program in the same set of proteins analyzed in this study, depicted in Figure S3. In this case we aim to test the significance of obtaining lower stabilities for experimentally determined unstable regions or for LIPs in comparison to stable protein regions, respectively, see bottom half of Table S2. All the statistical calculations were implemented using the R statistical package [57].

## Supporting Information

Figure S1 **Conservation of the polarity and packing density profiles in the flavodoxin family.** The polarity and packing density profiles of some members of the Flavodoxin family with known structure are shown. The different members of this family were structurally aligned and the polarity and packing profiles obtained were superposed taking the structural alignment as template. The proteins analyzed were (PDBID: 1AHN), (PDBID: 1FUE), (PDBID: 1OFV) and (PDBID: 1FTG), the rmsd of the alignment is 3.20 Å.
(EPS)

Figure S2 **Conservation of polarity profiles in representative folds in SCOP (Structural clasification of proteins) database.** **A)** SCOP class α/β (fold TIM α/βbarrel), **B)** SCOP class α+β (fold Lysozyme-like), **C)** SCOP class all α (fold Cytochrome c) and **D)** SCOP class all β (fold Immunoglobulin-like β-sandwich). The PDB IDs of the proteins analyzed are indicated and a cartoon representation of each protein fold is shown. The apoflavodoxin profiles shown in Figure 2A represent an additional example of polarity ratio conservation in class α/β (fold Flavodoxin-like).
(EPS)

Figure S3 **Anti-correlation of the polarity ratio and packing density of buried interfaces.** The packing density of buried interfaces calculated for apoFlavodoxin is plotted against their polarity (polar/apolar buried surface, see methods). The resulting dispersion plot shows a propensity of high polar interfaces to be less packed than other interfaces of the protein, resulting in an anti-correlation between these properties. The correlation coefficient of the adjust line is *R = 0.52*.
(EPS)

Figure S4 **Conservation of packing density profiles in representative folds in SCOP (Structural clasification of proteins) database.** **A)** SCOP class α/β (fold TIM α/β barrel), **B)** SCOP class α+β (fold Lysozyme-like), **C)** SCOP class all α fold Cytochrome c) and **D)** SCOP class all β (fold Immunoglobulin-like β-sandwich). The PDB ids of the proteins analyzed are indicated and a cartoon representation of each protein fold is shown. The apoflavodoxin packing density profile shown in Figure 2 represent an additional example of packing density conservation in class α/β (fold Flavodoxin-like).
(EPS)

Figure S5 **Residue stability estimations using COREX.** For each protein studied in this report using our methodology we also calculate the local stability using this alternative procedure. As described in methods, for each protein we first generated the structure ensemble used in the calculations, then determined the entropy-weighting factor before obtaining the corresponding stability constants. The residue stability obtained are plotted in this figure in the following order: **A)** apoflavodoxin, **B)** cytochrome c, **C)** α-lactalbumine, **D)** indole-3-glycerol phosphate synthase and **E)** barnase. In each case the unstable regions determined experimentally are colored in light grey.
(EPS)

Figure S6 **Sequence conservation of LIPs in three protein families.** Each protein family was aligned as described in methods and the corresponding structural alignments were processed using JOY to obtain a detailed representation of the alignment including structural information. In the alignments the solvent accessible residues are in lower case and buried residues in uppercase. We also show in the top line of each alignment fifty-column block the qualitative representation of conservation and in the bottom line we include the histogram with the quantitative estimates of conservation scores in a given position as calculated by CLUSTAL. With these data, the average conservation scores for alignment columns (corresponding to a given aligned residue) have been calculated for the complete alignments, for the experimentally determined unstable regions, and for the LIPs. We report the PDB codes of the proteins aligned, color in light grey the experimentally unstable regions, and remark the LIPs with a light blue line. **A)** Structural alignment of Flavodoxin family (the identity percentage (PID) is 34%). The unstable regions 87–108 and 118–152 are highlighted. The average column conservation score is 24%. However, the average column conservation score for buried and exposed residues in LIPs are 29 and 9%, respectively, and for buried and exposed residues in the experimentally unstable regions are 30 and 10%, respectively. **B)** Structural alignment of the Cytochrome c family (the PID is 47%) showing the 40–57 flexible region. The global average column conservation score is 33%, while the average column conservation scores for buried and exposed residues in LIPs are 49 and 30%, respectively, and for buried and exposed residues in the experimentally unstable regions are 42 and 25%, respectively. **C)** Structural alignment of α-Lactalbumin family (PID 50.4%) with the unstable region 40–80 in grey. The global average column conservation score is 43%, while the average column conservation scores for buried and exposed residues in LIPs are 73 and 41%, respectively, and for buried and exposed residues in the experimentally unstable regions are 70 and 44%, respectively.
(EPS)

Table S1 **Estimations of solvent accessibilities for a group of proteins.** From a set of 19 proteins from different folding families, different sizes and sharing less than 20% of sequence similarity we obtained the polar and apolar solvent exposed areas in the folded state (columns 3–5) and in the unfolded ensemble (columns 9–11) as described in (28). We also estimated the polar and apolar buried surface in the core of the folded state (columns 6–8). The averages ± SD of the ratio of polar and apolar areas for the three states of each protein are indicated in bold characters in the bottom line of the table.
(EPS)

Table S2 **Statistical significance of interfacial polarity and of COREX stability estimates.** We show the results of a one-sided Mann-Withney-Wilcoxon test performed on the interfacial polarity and the residue specific stability profiles obtained with our methodology and COREX, respectively. In the first case the alternative hypothesis *H1* test the significance of obtaining higher polarities in unstable segments determined experimentally (column: Unstable regions) and in the segments corresponding to our definition of LIPs (column: LIPs). For the stability estimates obtained with COREX the alternative hypothesis *H1* test the significance of obtaining lower stability values in the same protein segments described above. The confidence interval was set to ℘<0.05 in all cases.
(EPS)
