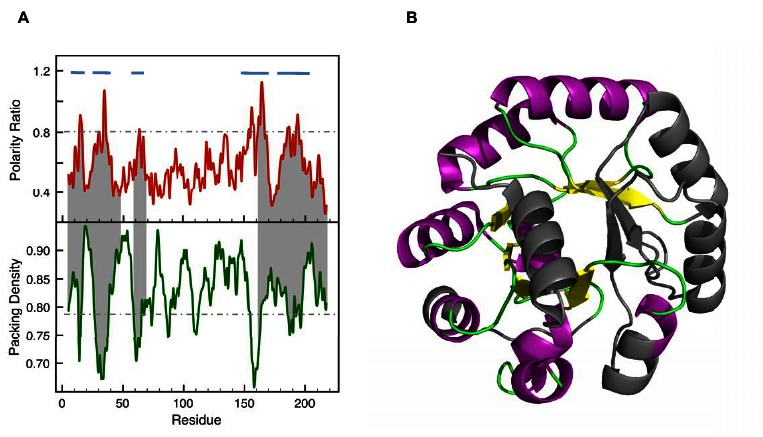# Correction: Protein Dynamics Governed by Interfaces of High Polarity and Low Packing Density

**DOI:** 10.1371/annotation/ba19c84a-4f59-46de-88fb-04a5f8222585

**Published:** 2013-05-16

**Authors:** Vladimir Espinosa Angarica, Javier Sancho

There was an error in Figure 4. The correct version is available here: 

**Figure:**